# Combinatorial Control of Gene Expression

**DOI:** 10.1155/2013/407263

**Published:** 2013-08-27

**Authors:** Soumya Bhattacharjee, Kaushik Renganaath, Rajesh Mehrotra, Sandhya Mehrotra

**Affiliations:** Department of Biological Sciences, Birla Institute of Technology and Sciences, Pilani, Rajasthan 333031, India

## Abstract

The complexity and diversity of eukaryotic organisms are a feat of nature's engineering. Pulling the strings of such an intricate machinery requires an even more masterful and crafty approach. Only the number and type of responses that they generate exceed the staggering proportions of environmental signals perceived and processed by eukaryotes. Hence, at first glance, the cell's sparse stockpile of controlling factors does not seem remotely adequate to carry out this response. The question as to how eukaryotes sense and respond to environmental cues has no single answer. It is an amalgamation, an interplay between several processes, pathways, and factors—a combinatorial control. A short description of some of the most important elements that operate this entire conglomerate is given in this paper.

## 1. Introduction

The orchestration of various biological processes with high fidelity requires a precise control over temporal and spatial expression of genes. The regulation of gene expression in eukaryotes can occur at various steps, namely, transcription, m-RNA splicing, translation, and Posttranslational modifications.

Transcriptional control can be achieved at any of the various stages—initiation, elongation, and termination. Initiation is marked by the assembly of the preinitiation complex at the promoter of the corresponding gene. The Preinitiation complex, comprised of RNA polymerase II and its auxiliary components (TFIIA, TFIIB, TFIID, TFIIE, TFIIF, and TFIIH) that bound to the core promoter, can only initiate basal levels of transcription [1]. Proper gene expression requires the presence of sequence-specific DNA binding factors called regulators.

Some regulators act by increasing (enhancers) or decreasing (repressors) the rates of transcription by stabilizing or destabilizing the interactions of the polymerase with the DNA or the auxiliary components, respectively. Others act by modifying chromatin structure. Chromatin structure greatly affects transcription in eukaryotes as it determines the accessibility of different regions of DNA to the incoming binding factors. Some regions are tightly coiled and are lesser accessible to the transcriptional machinery, while others are loosely coiled and easily accessible. A class of regulators called chromatin modifiers (CM) affects the rate of transcription initiation by increasing or decreasing accessibility of various regions. CMs function by two mechanisms—chromatin remodelling or by facilitating histone tail modifications. Both of these processes are ATP intensive and involve either increasing or decreasing interactions between the parent DNA and the histone cores [2]. 

Another class of molecules called coregulators binds to regulators and affects transcription by either stabilizing or destabilizing interactions between regulators and the Basal-transcription machinery. Long noncoding RNA molecules constitute a special class of coregulators because of their proteinaceous nature. The latter is also the reason why the study of these molecules has been of great interest in recent times. Some of the findings will be presented in a later section.

Transcriptional elongation involves the actual process of m-RNA synthesis from parent DNA strand. It has acquired great interest in the recent times because of its possible role as a checkpoint for control of gene regulation. Transcriptional pausing is a very common feature in elongation. Pausing occurs due to interactions between the RNA polymerase molecule and the m-RNA molecule, which may assume secondary structures such as hairpin loops and lead to consequent transcriptional attenuation. The latter may lead to premature termination and may consequently result in alternate products, thereby, affecting gene expression. Certain in vitro simulation studies have shown that pausing of the polymerase is briefly followed by microbursts in m-RNA yield, which was explained by the authors as a consequence of collision of two RNA polymerase molecules [3].

The primary m-RNA product may be subject to alternate splicing or preliminary RNA editing. Both of these processes may yield different translational precursors and, consequently, different protein products. The mature m-RNA transcript is finally translated into the corresponding protein product. 

Control at the level of translation is achieved by numerous mechanisms. One mechanism that has been extensively studied involves the role of small non-coding RNA molecules. These RNA molecules are majorly of two types—small interfering RNA (siRNA) and micro-RNA (miRNA). Both siRNA and miRNA molecules inhibit translation by two predominant mechanisms. siRNA molecules lead to the recruitment of RNA-induced silencing complexes (RISCs), which cut up the target mRNA molecules. On the other hand, miRNA molecules lead to the recruitment of microRNA-induced silencing complex (MISC), which physically blocks ribosomal translocation during translation. The role of small non-coding RNAs has frequently been noticed in the process of cell differentiation [4].

The final control checkpoint comes at the level of posttranslational modifications (PTMs). These affect the structure and consequently the activity, stability, and functioning of the protein. PTMs are hence critical to the cellular role of proteins. PTM in histones such as methylation, acetylation and ubiquitination at specific residues critically affects the conformational states of chromatin in the cell by altering the stability of histone-DNA interactions [2].

Although numerous checkpoints exist, complex controls of gene expression are noticed almost extensively at the level of transcriptional initiation. Transcriptional Initiation controls are extremely diverse and complex and display extreme variability in terms of the number of types of underlying molecular signals. The reasons supporting the latter are presented below.

The number of genes in an organism by far outnumber the numbers of transcription factors. For example, the human genome has about 20,000–25,000 genes. Each of these genes has multiple, unique temporal, and spatial expression patterns. Yet the number of DNA binding transcription factors is around 1850 [5]. The types of cis-regulatory elements are also dwarfed in comparison to the number of expression profiles of the entire genome. Hence, the question arises as to how a cell can perceive so many external signals and exhibit such a variety of gene expression with such a limited number of factors to work with. 

Considering the diversity of molecular signals that the transcriptional machinery has to interpret, it would be impractical for each regulator to have a unique target. On the other hand, the specificity of signals cannot be compromised.

Since each gene is under the control of more than one type and number of cis-regulatory elements, a combinatorial regulation enables the organism to have innumerable expression patterns even with a limited number of transcription factors. The present challenge before functional genomics is to understand how the different permutations of the same DNA binding factors alter individual gene expression.

This paper deals with some of those factors or points in transcription that allows the cell to manage its resources in such an efficient and brilliant manner and to elicit the necessary response. These have been divided into four levels of discussion—cis-regulatory modules, transcription factors, co-regulators, and long non-coding RNA-mediated control.

## 2. CIS-Regulatory Modules

Binding of DBTFs is sequence specific. While these sites are degenerate, they do have a certain level of consensus. Such sites, having a fixed consensus region and some variable regions are called cis-regulatory elements. The open question which exists is how cis regulatory elements which are similar in different promoters are not targeted in response to a particular cue. It seems that the process of elimination is much robust than the process of binding. Flanking sequences and chromatin state both contribute to this but still the mechanisms are not very well understood. There are regions on the DNA, where combinations of cis-elements occur in clusters. These cis-regulatory modules affect transcription, even if they are located far away from the target gene. Based on their impact on the activity of gene transcription, these are further subdivided into enhancers (if they increase the rate of gene transcription) and repressors (if they decrease transcription of target gene). They are similar to proximal promoters but a single cis-regulatory module can control the expression of different genes at different times. Cis-regulatory modules control the spatial and temporal expression of genes, independent of their distance and orientation relative to the promoter. Cis-regulatory modules are central to the combinatorial control of gene expression. Which cis-regulatory module would influence the transcription of a gene at a particular time is determined by the combination of DNA binding transcription factors (DBTFs) and co-regulators attached to a cis-regulatory module at that time. Cis-regulatory modules are like a lock with multiple key holes, where each key is a combination of different transcription factors. The combination of DBTF in turn determines the effect of a cis-regulatory module on gene transcription. Experiments done with a synthetic promoter element revealed that the activity of two binding sites switched from activators to repressors, when the combination of transcription factors changed [6]. 

The activity of cis-regulatory modules might also change when different subsets of cis-elements of the cis-regulatory modules are bound by transcription factors. Therefore, it is the number and organization of the cis elements that determine the activity and specificity of a cis-regulatory module. These parameters control the interactions between the bound transcription factors, which finally facilitate the recruitment of the transcription machinery. The importance of these parameters can be understood using the example of NF-*κ*B and virus inducible enhancer of the human interferon-*β* (INF*β*) gene. The transcription factor NF-*κ*B has many target genes. The combinatorial nature of cis-regulatory modules can be described by the observation that the number and type of cis regulatory elements or positive regulatory domains (PRD) present on the enhancer of the INF*β* gene influence specificity and the level of induction. The enhancer has PRD-I, II, III, and IV, recognized, respectively, by transcription factors NF-*κ*B, IFN-regulatory factor 1 (IRF-l), and activating transcription factor 2 (ATF-2)/c-Jun.

### 2.1. Number and Type of CIS Elements

**Figure 1:**
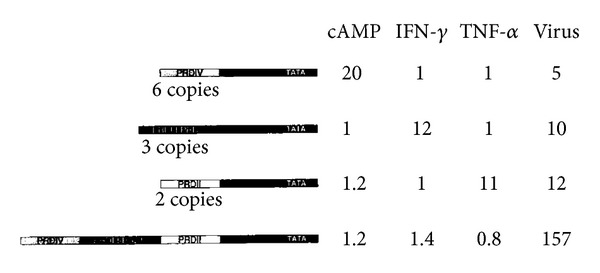
Comparison of the level of induction between the wild type and synthetic enhancers. Multiplication of cis-elements leads to a reduction in specificity and level of induction [8].

Combinatorial nature of enhancer activation is supported by the observation that mutation in any one PRD causes a marked decrease in the level of induction in response to viral induction. Synthetic promoters, each containing multiple copies of any one of the PRD, were tested for specificity. Promoters with multiple copies of the PRD-IV, III, and II showed nonspecific induction by cAMP, INF*γ*, and TNF*α*, respectively, whereas, the wild type enhancer is induced only by virus induction and not by these other factors (Figure 1) [7]. Thus, it can be said that the specificity of an enhancer not only depends on the cis elements present but also on their number.

The number of copies of any cis-element can also play a major role in determining its function. In the studies conducted in [9, 10], it was found that GT elements display different functions depending on their copy number. It was noticed that when a GT element occurred in a single copy, it led to induction of the *Pmec *promoter and an increase in transient gene expression in *Nicotiana tabacum.* On the other hand, two GT elements occurring in tandem led to the repression of the same promoter element [9, 10].

The number of cis elements is a consequence of segmental duplications over the course of evolution. It may be so that the importance of certain genes increased or decreased over the course of evolution, thereby, requiring an increase or decrease in its expression. This may have favoured certain duplication events over the others, as these may have produced the desired changes. 

### 2.2. Spatial Distribution of CIS Elements in an Enhancer

The spacing between the cis elements determines the interactions between DBTFs that bind to those elements. The spacing has to be proper keeping in mind the helical structure of the DNA, so that when the transcription factors are bound to the helical DNA they are able to engage in the proper interaction. Hence, the placement of cis elements might not reveal obvious interactions between transcription factors. 

In case of the INF*β* gene enhancer, the cis elements are placed very close to each other. In order to understand the importance of this arrangement, half or full helical turns were inserted between individual PRDs and their ability to induce reporter genes was studied [8]. When half helical turns (6 bp) were introduced between PRD-I and II, the level of induction fell drastically. However, activity was fully restored when a full helical turn (10 bp) was inserted between the two domains, as this reestablished the relative position of the binding sites on the helical DNA. Similar results were obtained when bases were inserted between other pairs of PRD [8].

In another study, it was hypothesized that the overlap of REs plays an important role in the assembly of the INF*β* gene enhanceosome complex. Simulation studies showed that the binding of the TF dimers introduced local conformational changes in the DNA, which led to the proposition that the specific overlap of binding sites on the DNA affected the enhanceosome binding cooperativity in the INF*β* gene. The same study also proposed that the order of cis-elements is very crucial to the activity of cis-regulatory elements. Reversal of the order of REs may destroy its functional significance by conferring opposite repressor activity [11].

In some cases, the nature of induction of promoters is governed by the spacing between the various cis-regulatory elements. Experiments conducted with synthetic promoters constructed with multiple ACGT elements separated by spacers of varying size highlight this concept. It was found that ACGT motifs separated by 5 bp showed specific induction by salicylic acid (SA) and partial induction by abscisic acid (ABA), while those separated by 25 nucleotides were specifically induced by ABA only [12].

These experiments highlight that the relative orientation of the regulators and consequent interaction between the regulators depend on the relative positioning of the cis-elements. These also reveal the existence of an important “chicken or egg” situation, which shall be addressed in a later section.

### 2.3. Positional Interdependence

The cis-regulatory elements contain core sequences, which are the binding sites for specific TFs. However, the neighbouring bases of these core-binding motifs also affect the binding of a TF to a cis-element. This is referred to as positional interdependence. Cis-element recognition by TFs becomes more specific due to this mechanism. This leads to a greater degree of control of gene expression. An example of this mechanism follows.

The Sox proteins bind to the core-DNA motif TTGT. Studies have shown that positional interdependence affects the binding affinities of different members of this family. For instance, in the sequence X_6_YTTGT_11_, if Y is a T, Sox4 has a high affinity when X is a C. Nevertheless, if nucleotide Y is an A, then A is preferred in the X position [13]. This indicates that the binding of Sox4 is affected by the flanking sequences to the core-binding motif. 

In silico analysis of the promoters of *Arabidopsis *revealed that spacers, which start with a G and end with a C, irrespective of their lengths, separate cis-ACGT motifs. The flanking of ACGT elements by C and G nucleotides is proposed to be extremely important for the binding of the TFs to these motifs [14].

### 2.4. Synergistic Interactions

Sometimes, different gene expression profiles are obtained based on the net effect, which is a product of multiple cis-element-TF interactions. This means that the individual effects of TF binding to different cis-regulatory modules interact with each other to produce the observed expression profiles. The presence of both AACA elements and ACGT motifs, each separated by different spacer lengths, regulates the expression patterns from the Protein Phosphatase-2C- (PP2C-) like promoter in *Arabidopsis thaliana.* The AACA motifs lead to repression of transcription while presence of ACGT elements increases the rate of transcription. In the 500 bp and 900 bp deletion constructs, it was observed that AACA element-mediated repression leads to an overall dip in expression profiles even though ACGT motifs were present [15].

Gene expression studies done on synthetic promoter constructs containing a variety of cis-regulatory motifs showed that the same cis-element could act both as an activator (when present alone) or could act as a synergizing agent that interacts with the other heterologous or homologous motifs to produce combinatorial effect on the transcription of the gene of interest. This study showed that such constructs showed a dip in the expression of the gene when TFs specific to one or more specific motifs were titrated away. This indicates that when present together, the different cis-regulatory modules act synergistically [16].

The existence of Synergistic interactions implies a need for coevolution of the different interactions that complement each other's function. The identification of domains where such interactions exist can throw light on some novel evolutionary signatures that could be of great interest in the field of molecular evolution.

## 3. DNA Binding Transcription Factors (Transacting Elements)

The Preinitiation complex, comprising of RNA polymerase II and its auxiliary components (TFIIA, TFIIB, TFIID, TFIIE, TFIIF, and TFIIH) that bound to the core promoter, can only initiate basal levels of transcription [1]. Proper gene expression requires the presence of sequence-specific DNA binding factors called regulators. Based on the type of DNA binding domain, regulators have been classified into several families—basic leucine zipper (*b*ZIP), cysteine rich zinc finger, helix-loop-helix (HLH), homeobox, and so forth. In addition to the DNA binding domain, regulators have a separate domain that is required to stimulate transcription. 

### 3.1. Spatial Orientation of Half Sites Adds Specificity to Transcription Factor Binding

 Most regulators are dimers and interact with the DNA at two sites, which means that their TFBSs are composed of two half sites. Just the mere presence of two half sites does not ensure binding and proper functioning of a regulator. The spatial arrangement of the half sites dictates binding specificity and regulatory function of regulators and thus aids in selective and combinatorial regulation.

The point of spatial orientation adds an interesting chicken or egg situation in evolution. It leads to rather interesting questions pertaining to which was a consequence of what—whether TF binding orientation was a consequence of a given spatial orientation of cis regulatory modules or if the spatial distribution of cis-elements is a consequence of specific TF binding properties.

### 3.2. Binding Strength of Regulator Influences Its Function

Regulators can bind wherever the consensus sequence is present but the variable part of the TFBS can influence the regulatory outcome, thus allowing the same regulator to fulfil different roles at different places. The binding strength of the regulators is affected by these variations. This phenomenon is evident during the developmental stages of Drosophila. The initiation of the formation of mesoderm, neuroectoderm, and dorsal ectoderm is caused by the varying levels of target gene expression by a transcription factor called dorsal morphogen (dl). This varied expression pattern is caused by a dl gradient. In the ventralmost regions (mesoderm), dl levels are high. Yet target gene expression is low, whereas in the ventrolateral region (mesoderm and mesectoderm), the expression of target gene is high in spite of intermediate levels of dl. This aberration has been attributed to the fact that the binding sites of dl in the mesoderm are low affinity binding sites, while the ones in the ventrolateral region are high affinity binding sites (GGG(A/T)nCCC, where n can be 4 or 5 A or T residues). Deviation of one nucleotide from the optimal core reduced the affinity almost five folds [17].

These observations lead one to speculate that in some cases, conservation of variable domains in the TFs might be of greater importance than that of the conserved domains themselves. Further studies aimed at understanding these changes might be helpful in weaving out some patterns that govern evolutionary changes in these variable domains.

### 3.3. Specific Dimerization Affects Binding Specificity and Imposes Tighter Control

Transcription factors found in nature occur as dimers. Dimerization can produce two types of dimers—homodimers and heterodimers. Heterodimerization involves dimerization between two different monomers. The benefit of producing more than one type of monomers is the greater diversity that can be obtained in the dimer products. Sometimes, heterodimerization optimizes the resultant combination in terms of the binding strength. This means that the heterodimer is better in terms of both binding specificity and affinity, as compared to either homodimers. This could be the result of each monomer cancelling out the negative binding attributes of the other.

Let us consider the examples of the ATF-2/c-Jun and IRF-3/IRF-7 heterodimers. These heterodimers belong to the class of TFs that bind to the INF*β* enhanceosome. ATF-2 and c-Jun are bZIP TFs that have different DNA binding specificities. The c-Jun TF has a lower binding specificity as compared to ATF-2. Some studies predict that the DNA sequence motifs and the c-Jun/ATF-2 dimer are designed such as to optimize the combination and enhance DNA binding specificity of each constituent monomer and cooperativity between neighbouring partners [11].

A similar situation exists in the case of the IRF-3/IRF-7 dimer. IRF-3 homodimers are characterized by very few interactions between the monomer DNA binding domains. This implies that the IRF-3 homodimerization is a wasteful process. It is also noticed that the IRF-7 binding to two sites is stronger than the corresponding affinity of IRF-3. It is hence noticed that the IRF-3/IRF-7 is much more stable than the IRF-3 homodimer. The heterodimer also seems to enhance the IRF-DNA interactions in the absence of other proteins [11].

### 3.4. Sequence Specific Alternate Conformation of Regulators Can Function Differently

**Figure 2:**
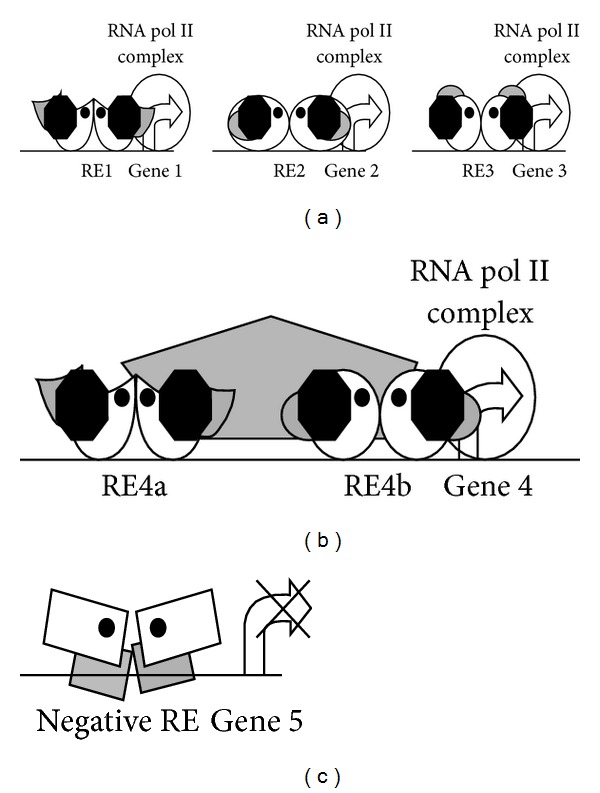
Different roles fulfilled by the same transcription factors. Steroid receptors (white shapes) bind to their response elements (REs) after being activated by their ligands (black dots). (a) Binding to different REs recruits a common cofactor and an element-specific cofactor (black octagon and grey shapes, resp.) (b) Binding to adjacent RE recruits a bridging factor. (c) Binding to negative REs recruits corepressors [18].

Minute deviation in the TFBS sequence is also known to cause conformational changes in the DNA binding domain of the regulators. This in turn facilitates the creation of a new platform, which can recruit various cofactors and even change local chromatin structure. This has been elucidated in case of various steroid receptors that also function as transcription factors. This type of allosteric effect of the TFBS greatly increases the possible varieties of gene expression (Figure 2) [18].

### 3.5. Posttranslational Modifications Of TFs Are Crucial Determiners of Their Specificity

The posttranslational states of transcription factors affect the functionality of the transcription factors. Scientific literature evidences of various posttranslational events such as acetylation, phosphorylation, and methylation and their effects on the functions of different classes of TFs have been recorded. The High-Motility Group Box (HMGB) family of proteins posees a good example of the same.

The HMGB class of proteins has significant roles in DNA maintenance and recognition processes. They are seen to possess DNA binding and DNA bending functions. The post-translational modifications affect these functions of the HMGB proteins. In bovine HMGB1 proteins, acetylation of Lys 3 increased the former's affinity for UV-damaged DNA [19], while that at Lys82 in the linker region also affected the proteins' DNA bending functions [20]. Similarly, phosphorylation of acetylated HMGB1 affected its DNA-end joining capabilities without affecting its DNA binding affinity [21].

One method of control of gene expression is by regulating of availability of TFs. In the HMGB1 proteins, it has so been found that the post-translational state of these proteins affects their cellular location [22]. Such coupling of mechanisms leads to tighter regulation of gene expression. 

### 3.6. Chromatin Structure and Cofactors Impart Tissue-Specific Behavior of Transcription Factors

Chromatin structure often prevents interaction of the transcription machinery with the promoter of certain genes and hence acts as a repressor. In the presence of tissue-specific cofactors, some transcription factors have the ability to modify chromatin structure and thus allow tissue-specific expression of certain genes. This involves ATP-dependant noncovalent modification of local chromatin structure and addition or removal of covalent groups from histone tails.

MyoD is a member of the myogenic basic helix-loop-helix family of regulatory factors (MRFs) and is essential for the induction of muscle-specific genes like myogenin, which leads to myogenesis during embryogenesis. This factor is indispensible for commitment to the skeletal muscle lineage [23, 24]. MyoD also induces the expression of genes like pRb, cyclin D3, p21, and so forth that are not muscle-specific and are expressed in all cells.

 It was found that MyoD-mediated activation of muscle-specific genes is heavily dependent on the SWI/SNF enzyme which is an ATP-dependant chromatin remodelling complex [25, 26]. On the other hand, the absence of the SWI/SNF complex did not significantly vary the expression of MyoD-induced global genes [25, 27]. These studies were done using cells that were dominant negative for SWI/SNF. It was also discovered that the recruitment of the SWI/SNF complex requires the promoters of the muscle-specific genes to be hyperacetylated [28]. In cells that are not destined to be muscle cells, the lack of this acetylation pattern in the muscle-specific genes ensures that MyoD-mediated expression of these genes does not take place.

This example elucidates that acetylation pattern; chromatin structure and its remodelling in part dictate tissue-specific behaviour of transcription factors.

## 4. Coregulators

The activity of transcription factors and hence the different patterns of gene expression in different tissues are controlled by another class of molecules, known as the coregulators. These molecules greatly increase the variety of responses that can be elicited by a limited repertoire of transcription factors. Coregulators do not typically have DNA binding activity, rather they exhibit protein-protein interaction and bind to the DNA bound transcription factors. Each type of cell has a specific set of co-regulators and this plays a major role in determining the response to a particular signal. A transcription factor bounded to a specific DNA sequence can act as either an activator or a repressor depending upon the coregulator associated with it. 

Coregulators can perform various functions and can be broadly classified on that basis. A class of coregulators can modify histones making certain genes accessible (coactivators) or inaccessible (corepressors) to the transcription machinery. For example, p300 and CBP are powerful histone acetyltransferases (HATs) and hence they act as coactivators, whereas corepressors like *NcoR* have histone deacetylase activity and play a role in gene silencing. Another class of co-regulators binds to transcription factors and is involved in recruiting RNA polymerase and the core transcription machinery. This includes members of the TRAP/DRIP/Mediator/ARC family.

Finally, a third class of co-regulators has ATP-dependent DNA unwinding activity and is essential for the transcription of certain genes. The SWI/SNF complex belongs to this group [20].

### 4.1. Coregulators Shift the TF Populations to Conformational States That Favor DNA Binding

**Figure 3:**
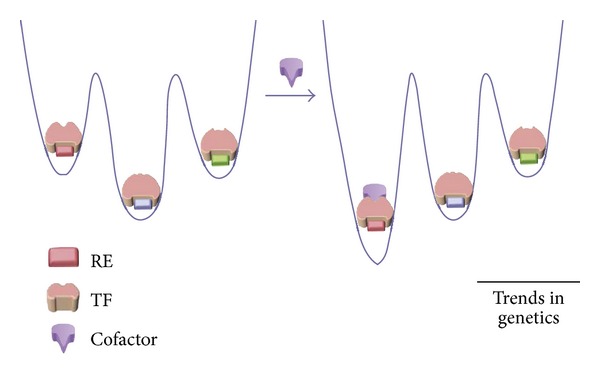
The figure shows the free-energy landscape of various TF-RE complexes (TF: pale pink; Res: blue, green, and pink/red; cofactor: purple). The binding of the cofactor causes a population shift towards a particular TF-RE complex (*indicated by the arrow*). As is shown, the TF complex with blue RE is the most stable prior to cofactor binding. After this event, the complex with the red RE is the most stable interaction. By the mechanism stated herein, the binding of the purple co-factor shifts the populations in favour of the TF-red RE complex [31].

In many cases, TFs do not bind to the DNA even though the REs of TFs are freely available for binding. The reason for this is that the population of TFs present exists in conformational states that do not favour DNA binding. In such cases, co-regulators bind to the TFs prior to the DNA binding event. This shifts the populations to conformational states that are stabilized by binding with a specific RE sequence (Figure 3) [29].

NMR studies of p53 binding to Apoptosis stimulating protein of p53 (ASPP) are a testament to this mechanism [30]. While p53 complex with ASPP-1 or ASPP-2 stimulates apoptosis, that with iASPP inhibits apoptosis. According to this mechanism, the binding of ASPP-1 and ASPP-2 shifts the populations of p53 in favour of the conformational states that are stabilized by binding to the RE that promotes transcription of proapoptotic genes. Similarly, the binding of iASPP leads to the increase in the numbers of those conformational states that favour binding to REs that repress transcription [31].

### 4.2. Functional Flexibility of Coregulators Is the Key to Combinatorial Control of Gene Expression

**Figure 4:**
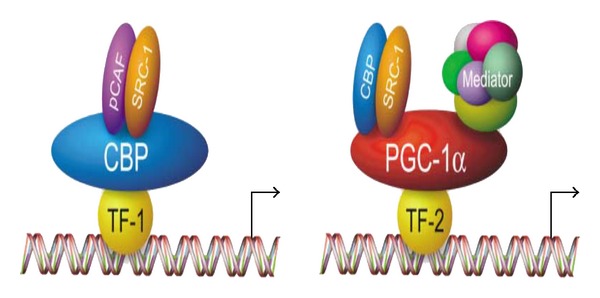
Flexible Nature of coactivator Proteins. CBP binds directly to transcription factors and recruit the secondary co-regulators pCAF and SRC-1, all of which have HAT activity. CBP also functions as secondary co-regulators to PGC-1 which directly binds to another transcription factor [23].

Co-regulators have the ability to recruit secondary co-regulators that further diversify the function of a particular transcription factor. For example, the co-activator CBP can bind to transcription factors as well as recruit coactivators like SRC-1 and pCAF, which have similar HAT activity. In addition to serving as a primary coactivator, CBP can act as a secondary co-activator to PCG-1 that binds to another transcription factor. Such varied and complex interplay of molecules having flexible nature gives cells the option of forming innumerable biological programs using a limited arsenal of transcription factors (Figure 4) [23].

### 4.3. Co-Regulators Control Tissue-Specific Behaviour of DNA Binding Transcription Factors (DBTFs)

Some DBTFs have binding sites on the promoters of a variety of genes that they induce in different tissues. Along with this, they also induce certain tissue-specific genes in some cells and not in the others. This discretion can be, in part, attributed to the presence of certain co-regulators in some tissue as against the others. This phenomenon is observed in the following cases.

#### 4.3.1. Oct-2 and OCA-B

Expression of immunoglobulin (Ig) genes requires octamer binding proteins (Oct) to bind to the promoters of Ig genes. Oct-1 is expressed in many cells; however, Oct-2 is mostly restricted to B cells. Therefore, it was hypothesized that Oct-2 is the factor that enables B cells to produce Ig molecules. However, Oct-2 alone was unable to perform this function in cell free systems and in transfected cells. Biochemical purification of Oct-1 and 2 revealed a co-activator, OCA-B which has chromatin modifying enzymatic activity [32]. It recruits other factors like PC-4 and TAFII-105 to the transcriptional complex, anchored to the DNA by Oct. OCA-B is highly specific to B cells only, and in its absence, mice B cells cannot produce Ig molecules [33–35]. Therefore, it can be concluded that the co-activator OCA-B controls B cell-specific Ig gene induction by Oct transcription factors.

#### 4.3.2. SRF and Myocardin

Serum response factor (SRF) binds to the promoters of many muscle genes and induces their expression. SRF also has binding site in the promoters of many nonmuscle-specific genes. SRF is also found in the cells and tissues other than muscle. Therefore, there must be a molecule that is associated with SRF, which imparts muscle-specific function. From in silico data, it was evident that myocardin is one such molecule as it can interact with SRF via an SAP domain and it is found only in smooth and cardiac muscle cells [36]. Experiments revealed that myocardin enhances expression of smooth muscle cell markers *α*-SM actin and calponin [37]. Myocardin expressed in embryonic stem cells caused them to differentiate into smooth muscle cells [38, 39]. From all of this, it can be inferred that myocardin is the master regulator of smooth muscle cell development and is responsible for the tissue-specific function of SRF in smooth muscle cells during development.

Largely, transcription is controlled at the co-regulator level especially through co-activators. Even the co-regulators themselves can be a target for developmental or physiological signals. The combinatorial control of gene expression cannot be fully appreciated without considering the co-regulators. Transcription factors and their associated co-regulators form complexes that control transcription. By the different permutations of co-regulators, even a small number of DBTFs binding to a limited number of cis regulatory elements can form a system, which can execute the innumerable gene expression profiles that are necessary for sustaining eukaryotic organisms. Furthermore, the biological system gains additional control of gene expression by modifying the co-regulators via different cell signalling pathways, which allow the regulators to target different subsets of co-regulators at different genes or in different cell types.

## 5. Long Noncoding RNA-Mediated Control

**Figure 5:**
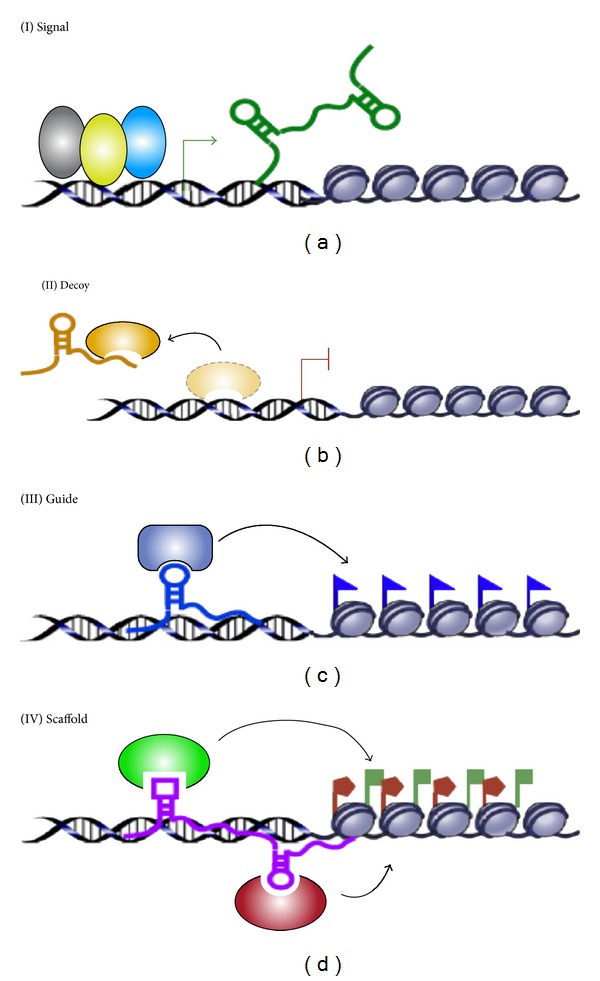
lnc-RNA molecules can function via multiple mechanisms as shown here. (I) Lnc-RNAs act as signalling molecules to other molecules that are involved in chromatin remodelling. (II) lnc-RNAs act as decoys and divert the effector molecules away from target DNA. (III) Lnc-RNAs guide the effectors to their respective target sites. (IV) Lnc-RNAs may function as a binding substratum for multiple protein molecules which act on the target gene sequence after assembly [40].

Most of the genome consists of non-coding DNA regions. Recent research is indicative of the significance of the long non-coding RNA (lnc-RNA), which is the byproducts of transcription of these regions. Lnc-RNA molecules are 200 nt or greater in length and are seen to play an important role in transcriptional control of gene regulation. They are poorly conserved through the course of evolution, unlike other conserved RNA regulators like micro RNAs, siRNAs, and so forth that function by means of base-pairing mechanisms. There is no concrete mechanism to explain the mechanism of control exercised in the case of lnc-RNAs, but four distinct mechanisms have been observed (Figure 5) (reviewed in [40]).

### 5.1. Lnc-Rnas Might Act as Signals for Recruitment of Chromatin Modifying Machinery

Lnc-RNAs, as proposed by this mechanism, might act as signalling molecules for the recruitment of other chromatin remodelling complexes thereby regulating target gene expression. The signalling can be the product of the regulatory function of the RNAs or because of the very event of transcription of lnc-RNAs. The use of RNAs as signals saves the cell the time involved in translating the RNA precursors into regulatory proteins [40]. The repressive histone modifications observed in the regulation of *Kcnq1 *and *Igf2r* imprinted gene clusters in mouse placenta are mediated by accumulation of lnc-RNAs near the target promoters [41]. 

Such mechanisms are observed in plants also. For example, the vernalization-mediated epigenetic control of the Flowering Locus C (FLC) in *Arabidopsis thaliana *is mediated by lnc-RNAs called Cold Assisted Intronic Non-Coding RNA (COLDAIR). The latter physically associates with PRC2 complexes at the FLC locus and mediates the chromatin modifications that lead to transcriptional repression. The PRC2 complexes are important in carrying out the epigenetic changes observed [42].

### 5.2. Lnc-RNAs Might Function as Decoys

In other instances, lnc-RNAs have been found to impersonate target DNA and hence interfere in the assembly of the regulatory transcriptional machinery. Such a mechanism has mostly been associated with transcriptional repression by means of misleading the effector molecules [40]. An example of this mechanism can be found in the regulation of the DHFR (human dihydrofolate reductase) gene. Transcription of lnc-RNAs from the upstream minor promoter leads to interference in the binding of TFIIB TF at the major promoter due to interaction between the former and the latter [43].

### 5.3. Lnc-RNAs May Guide the Effector Molecules to Their Target Site

In many cases, lnc-RNAs may act as guides and aid in directing the effector molecules, which may be cis-regulators, transacting proteins, or TFs, to their respective binding sites [40]. These are mediated through the structural modifications of the epigenome accompanying RNA transcription and RNA-DNA interactions (reviewed in [44]).

 Such a mechanism is prevalent in X-chromosome inactivation (XIC). An important step in *XIC* is the recruitment of polycomb repressive complex 2 (*PRC2*) which is achieved through *PRC2* binding to *RepA*, which is an lnc-RNA transcribed from the 5′end of the Xist gene [45]; that is, *RepA* guides *PRC2 *to its target site. 

### 5.4. Lnc-RNAs May Function as Scaffolds for Recruitment of Effectors

Sometimes, lnc-RNAs might act as scaffolds or recruitment sites of multiple proteins that are to be targeted to a particular gene. By doing so, they aid in bringing together multiple proteins in both time and space [40].

The HOTAIR lnc-RNA is one such example. This stretch of lnc-RNA contains two sites—a 300 nt stretch in its 5′end and a 700 nt stretch towards its 3′end. The former is the binding site for PRC2 proteins, while the latter forms the binding site for LSD1/CoREST/REST complex [46]. Both of these complexes catalyse histone tail modifications that repress gene transcription. By acting as a recruitment site for both of these complexes, the HOTAIR molecule can mediate gene silencing by means of multiple mechanisms [40].

 The HOTAIR molecule is unique in that it can both act as a scaffold as described above or as a guide that aids in targeting of PRC2 to transregions of the genome. Such integrated approaches have also been found in case of other lnc-RNA molecules, whose functions are implicated in other cellular events [40]. This last point highlights the presence of integrated mechanisms, emphasizing the notion of combinatorial control.

## 6. Conclusions

The cell performs an amazing feat interpretation constantly varying external signals and keeping with an ever-changing pattern of gene expression. It does so with an intricate network of mediators and signalling molecules; those are integrated together by using an equally complex system of signal transduction. 

The plethora of internal signalling required for gene expression is created by the cells by cleverly utilising a comparatively limited reservoir of molecules. The principle of combinatorial control of gene expression that is addressed in this paper sheds light on the mechanisms used by the biological systems to achieve tight regulation of the entire process. The cells use the limited amount of resources available at their disposal, in the most innovative and economic ways to achieve the same. It points to a situation where the cells utilize a handful of molecules in various permutations and combinations to accomplish the startling task of constantly switching on genes and switching them off according to their needs. 

The controls of gene expression show complex and diverse patterns and are subject to various constraints such as factor availability. It is also noticed that although one tries to map the entire process by a linear series of events, the various steps are very closely knit together (reviewed in [47]). In such a scenario, it is virtually impossible to isolate a unique mechanism that governs the entire process. The aim of functional genomics is to study the different mechanisms that are prevalent in some common biological systems and device models that encompass the different observations. These models can then be used in various other fields of research application. This paper is an attempt to cover some of these mechanisms observed at the level of transcription initiation.

The cis regulatory modules, regulators, and coregulators are at the foundations of these mechanisms. Emerging discoveries regarding the roles of lnc-RNAs point to newer biochemical pathways and emphasise the notion of combinatorial control. These studies point to a new era, which might look to alter gene expression profiles by increasing the concentrations of these RNA products within the cell. Each of these elements shows a variety and flexibility of function depending upon their conformational states. Additionally, the situation is made more interesting by availability of various regulators and coregulators, which aids in the further selection of the combination required. The use of regulators and co-effectors with less than 100% binding efficiencies in systems, whose performance is optimal for satisfying the cell's needs, is a testament to the efficiency of the latter.

The use of such complex signalling cascades also gives the cell greater control over the process as it now contains more than one regulatory step. Such a high degree of control is indispensable for the cell. The holistic understanding of these mechanisms will aid in improving the techniques used in r-DNA technology, developmental biology, and so forth. Such improvement will aid in the development of applications that benefit humanity.
